# Female Preference and Predation Risk Models Can Explain the Maintenance of a Fallow Deer (*Dama dama*) Lek and Its ‘Handy’ Location

**DOI:** 10.1371/journal.pone.0089852

**Published:** 2014-03-05

**Authors:** Marco Apollonio, Fabio De Cena, Paolo Bongi, Simone Ciuti

**Affiliations:** 1 Department of Science for Nature and Environmental Resources, University of Sassari, Sassari, Italy; 2 Department of Biometry and Environmental System Analysis, University of Freiburg, Freiburg, Germany; Hungarian Academy of Sciences, Hungary

## Abstract

We tested the predictions of three models (female preference; hotspot; predator avoidance) on lek formation in the fallow deer population of San Rossore, Tuscany. We collected behavioural observations in two leks and radiotracking data on 67 deer over 7 years. Two deer sub-populations were present in the northern and southern sides of the area, respectively, the two sectors being delimited by a river and including one lek each. Predictions were tested for one lek (SG), located in the south-side where we set up our 7-year radiotracking program. Data from a second lek (FO, north-side) were used to test those predictions which imply the occurrence of multiple leks in the same population. We showed that the majority of females made one single visit to one lek, only during the rut. The lek was located outside areas of higher female traffic and home range overlap, and females increased home range sizes during the rut to reach it. Twilight routes of females never crossed the lek; instead, females walked atypical routes and at a faster pace to reach the lek and mate. The distance between the two leks was higher than the average diameter of female home ranges, and only one lek was present within female home ranges. Males reached the lek one month before the arrival of females, corroborating that lekking is a female-initiated process (females moving towards large clumped male aggregations) rather than a male-initiated process (males moving towards female hotspots). Our results supported the female preference model, and rejected the predictions of the hotspot model. Also, leks were located far from areas with higher predation risk, supporting the predator avoidance model. The position of lek SG resulted ‘handy’ at the sub-population level because of the optimal trade-off between travel costs for females to reach it and avoidance of human predators.

## Introduction

A lek is a clump of displaying males that females attend primarily for the purpose of mating [1]. Leks have been described in a wide array of animal taxa from arthropods to mammals (reviewed in [1]) but are rare in the latter, occurring only in few ungulate species [1]–[6]. As in many birds, ungulate leks can normally be found in traditional locations and are characterized by a skewed male mating success [1], [7]–[11].

A plethora of hypotheses have been proposed to explain why leks occur at traditional mating sites [1], [5], [12]–[16]. However, models that predict the formation of a lek without any specific reference to its spatial location cannot be easily tested if the lek already exists. For example, the hotshot model [17]–[20] predicts that females prefer to mate with an attractive “hotshot” male, usually surrounded by unattractive males that try to parasitize his attractiveness. This phenomenon could lead to the formation of a traditional lek site [17]–[20], but with no clues as to its position. Similarly, the black hole model ([14], also known as the female harassment model [21]) predicts that the sexual harassment by subadult males leads females to find refuge within a territory defended by an adult male, and adult males could have a higher chance to retain a harassed female when males are clustered in a lek. Once again, this model predicts the formation of a traditional mating site, but with no clues as to its spatial location. Both the hotshot and the black hole models could be excellent explanations on how leks initially form. Bradbury and Gibson [13] claimed that lek formation may be due to multiple factors (e.g., black hole, hotshot), but selective pressures should be responsible for the persistence of certain leks and the disappearance of others within the same population.

When leks are already formed, such as in our research, it is possible to test only those models on lek formation that include clear predictions about its spatial location; thus, we can test those models that would explain why a lek is favoured by individuals of a population and why the lek persists in that specific location. These models are: i) the female preference model [22], ii) the hotspot model [23], and iii) the predator avoidance model [11]. Here we aim to test these three models using data collected over 7 consecutive years in the fallow deer (*Dama dama*) population of San Rossore (Italy), where lekking has been documented since 1980s [8], [24]. Two different leks were active at the time of our research. Two deer sub-populations were present in the northern and southern sides of the area, respectively, the two sectors being delimited by a river and including 1 lek each. Predictions were tested for one lek (SG), located in the south-side where we set up our 7-year radiotracking program. Data from the second lek (FO, north-side) were used to test those predictions which imply the occurrence of multiple leks in the same population.

### The female preference model

According to this model, leks form because females prefer large clump of males due to higher mate choice opportunities [18], [22], [25], [26]. As a consequence, almost all females in a given population should choose to mate with males in a lek. The clustering of females would thus be determined by the clustering of males, and not vice versa. Within a spatial framework, females are expected to increase their home ranges to select a male from this male aggregation [19], [20]. Bradbury [26] and Bradbury & Gibson [13] suggested that female preference for larger leks would cause males to cluster until there is a single lek per population or per female home range. Leks should thus be spaced an average female home range diameter apart (*i.e.*, only one lek within a female home range), and each female should visit only one lek.

### The hotspot model

According to this model, leks forms in those sites (namely hotspots) where the probability for males to encounter females is high [13], [23]. Such hotspots could be located where female home ranges overlap [23]. Males would be expected to use such areas because of high female encounter rate, while females would also benefit because their travel costs to meet potential mates would be minimized. In this case, the clustering of males would thus be determined by the clustering of females, and not vice versa. In addition, the model predicts that females could visit more than one lek before breeding and, as a consequence, there should be more than one lek within a female home range [13], [26]. Leks are expected to be spaced by less than one average female home range [13], [26] and, females are not expected to increase their home range size to mate [13]. These predictions were suggested to help discriminate between the hotspot model and models that assume female choice for lekking males, *i.e.*, the female preference model [13], [26].

### The predator avoidance model

According to this model, leks form in areas where predation risk is reduced [27], [28]. Many authors have considered the benefits of mating in a lek for both sexes because of reduced predation risk due to dilution effect [1], [11]. For this reason, lek should be used by the majority of individuals in a population. So far, though, few researches have taken into consideration the position of the lek with respect to predator home ranges. In topi (*Damaliscus lunatus*), for instance, leks are located where the grass on the savannah is short and the risk of predations by lions may be reduced [29]. The same has been suggested for the Uganda kob (*Kobus kob thomasi*) [30].

The female-initiated process (*i.e.*, females moving towards large clumped male aggregations) described by the female preference model is in contrast with the male-initiated process (*i.e.*, males moving towards female hotspots) predicted by the hotspot model. Thus, mutually exclusive set of predictions can be listed for these two processes. Predictions derived from the predation avoidance model can be partly applied to both the hotspot model and the female preference model. Accordingly, we tested a full set of predictions of these 3 models (Table 1) using fallow deer behavioural and radiotracking data to shed light on the selective pressures that favour the persistence of a lek in a specific location.

**Table 1:** Predictions of three models on lek formation.

		Models' predictions		
	Field data (1997–2003)	Female preference model	Hotspot model	Predator avoidance model
**LEK VISITS (OCCURRENCE AND TIMING)**	**1.1** – Percentage of females that visited the lek during the rut^1^^,^ 3 ^,^ 4	All females	Only females that have at least a lek within their home range	All females
	**1.2** – Percentage of males that visited the lek during the rut^1^^,^ 5	All males	*No prediction*	All males
	**1.3** - Number of visits to the lek per female during the rut^1^	1 (repeated if mating does not occur)	Several visits	*No prediction*
	**1.4** - Number of leks visited by each female during the rut^1^^,^ 3 ^,^ 4	1	More than 1	*No prediction*
	**2** – Timing of male visits to the lek and marking behaviour of males before, during, and after the rut^1^^,^ 5 ^,^ 6	Males go to the lek in order to make visual and olfactory references for dominance well before the begin of the rut, i.e. *well before the appearance of females*	Males go to the lek in order to make visual and olfactory references for dominance well before the begin of the rut, *when females already use or cross this area*	*No prediction*
**RELATIONSHIP BETWEEN POSITION OF LEKS AND FEMALE HOME RANGES AND MOVEMENTS**	**3.1** - Number of leks within a female home range^4^	1	More than 1	*No prediction*
	**3.2** - Distance between two leks^4^	Higher than a female home range diameter	Lower than a female home range diameter	*No prediction*
	**4** - Home range sizes of females and their position with respect to the lek outside and during the rut^2^^,^ 3 ^,^ 4	Female home range sizes increase during the rut. Home range centers are far from the lek center	Female home range sizes do not increase during the rut. Home range centers are close to the lek center	*No prediction*
	**5.1** - Leks' location with respect to female deer movement outside the rut and during the rut^2^^,^ 3 ^,^ 4	Outside the area of higher female traffic and higher female home range overlap	In the area of higher female traffic and higher female home range overlap	All leks are located in the area of lowest predation risk
	**5.2** - Usual daily movements of females before, during, and after the rut^3^	Daily female movements do not cross the lek	Daily female movements cross the lek	*No prediction*
	**6** - Movements of females to the lek^3^	Atypical if compared to usual daily movements	Typical if compared to usual daily movements	Directed towards the area with low predation risk

Predictions of 3 models on lek formation related to field data collected in the San Rossore fallow deer population over 7 consecutive years. Two leks with more than 15 actively defended territories were present during the study.

Data sources:

1 *direct observations on leks;*

2 *discontinuous radiotracking of females outside the rut;*

3 *continuous radiotracking of females before, during, and after the rut;*

4 *discontinuous radiotracking of females during the rut;*

5 *discontinuous radiotracking of males during and outside the rut;*

6 *marking activities collected outside the lek before, during, and after the rut.*

## Methods

### Ethic statement

Deer captures performed by game keepers of the Estate were aimed to translocate deer into different Estates of Tuscany. These operations were targeted to reduce deer density, improve deer health and welfare, and limit vegetation over-browsing. Based on a research and management agreement between the University of Pisa (former insitution of MA & SC, 1997–2000), the University of Sassari (MA, SC, since 2000) and the administration of the San Rossore Estate, deer captures were approved by MA and SC, who were in charge of the wildlife management of the Estate during the whole study period (approval of deer capture and translocation operations are included in the official reports of the San Rossore estate; official reports # 1-14: 1997–2010;). Procedures were in accordance with all relevant Italian wildlife and animal welfare legislation – including regional (Regione Toscana) and provincial (Provincia di Pisa) major laws and rules on animal health and welfare. No specific permissions were required to capture deer to be fitted with radiocollars for this study, because such captures were part of the management operations in the Estate already approved by MA, SC, the authorities of the Estate, and of the Migliarino-San Rossore-Massaciuccoli Regional Park. The field study did not involve endangered or protected species, and this implied that approval from Institutional Animal Care and Use Committee was not required.

### Study area

This study was conducted in the San Rossore Estate (4,650 ha), central Italy (43°43′N, 10°19′E) which was mainly covered by pine and mixed deciduous woodland and, to a lesser extent, by wet deciduous woods, marshes, and meadows [31]. Cultivated areas (946 ha) were fenced and were not accessible to deer. Areas along the coast (dune vegetation, degraded coastal zone, and maritime pine woods) were not used by deer [31], [32]. The eastern sector of the Estate (namely the disturbed sector, 466 ha) was characterized by high human disturbance during the period of the study (*i.e.*, 1997–2003) [33]. Humans were the main predator of deer in this area [34], [35]. As a consequence of the different response to predation risk by sexes, the disturbed sector was used mainly by adult males outside the mating season and strongly avoided by females with their fawns [32]–[35]. Two traditional leks have been documented since the 1980s with at least 15 actively defended territories [35]: the lek of Stacca del Gatto (lek SG, south side of the study site), and the lek of Fossacci (lek FO, north side). Another historical lekking site (lek Macchia Capraia, MC) was present in the south side of the Estate until 1992 and was then abandoned by deer after habitat manipulation [7]. It was never used by deer during this research (1997–2003).

### Captures of deer

Deer randomly chosen for research were driven by 20–30 game keepers into circular corrals during winter from 1996 to 1999. Thirty-six bucks (>4 y.o.) and 31 adult females (>1 y.o.) were hand-caught, blindfolded, aged by tooth wear [36], ear tagged, fitted with Televilt VHF radiocollars (Lindesberg, Sweden), and finally released (see [34] for more details).

### Observation of lek activities during the mating season

**Figure 1:**
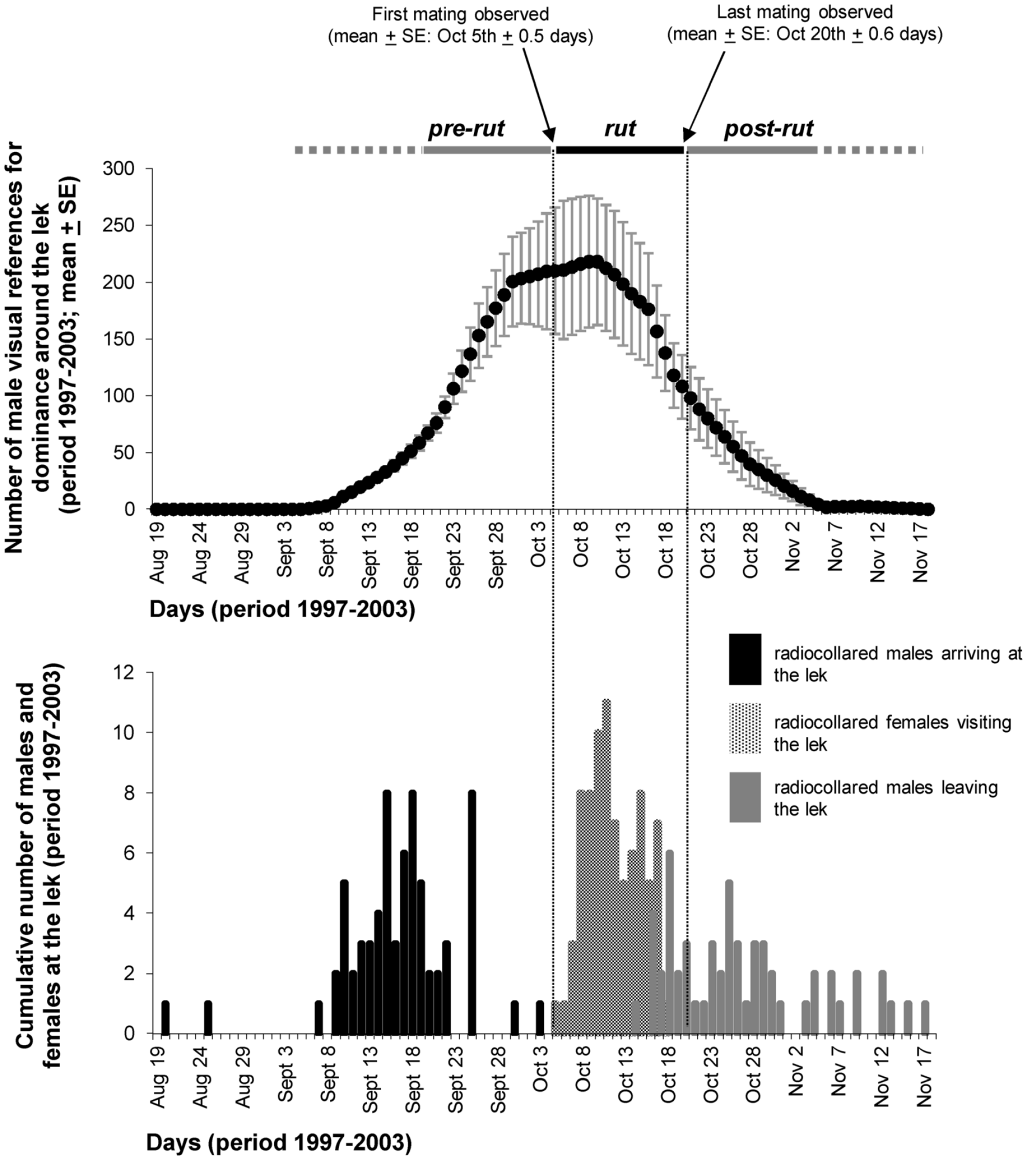
Timing of lek use by male and female fallow deer. *Upper panel* - Occurrence (mean ± SE) of male visual references for dominance (*i.e.*, male marking activities on the ground or the vegetation) recorded outside the lek from late August to mid-November (1997–2003). *Lower panel* - Number of radio-collared males that arrived at (black bars) or left (grey bars) the lek during the mating season (period 1997–2003). Nine males left the lek later than Nov 17^th^ and thus were not included in the figure. Number of radio-collared females that visited the lek during the mating season is indicated by shaded bars.

Direct observations of mating activities during 7 consecutive years (1997–2003) were performed from 3 camouflaged shelters along the borders of the 2 leks [35]. A minimum of 2 observers per shelter carried out direct observations using both binoculars (10×) and telescopes (30–45×). Continuous observations of lek activities (every day from dawn to dusk) began when territorial defence was first detected (late September-early October), and ended when defence ended (late October) [8], [35]. Observers of each lek were equipped with a VHF receiver and used to verify the presence of collared deer every 15–30 minutes. The rut (*i.e.* the peak of the mating season) was defined as the time between the first day (from 1997 to 2003: mean day ± SE = October 5^th^±0.5 days) and the last day (October 20^th^±0.6 days) on which copulations were recorded each year. Accordingly, pre-rut and post-rut were defined as the periods preceding or following the rut, respectively (Fig. 1). From this set of behavioural data collected within each lek (see [35] for more details), we used the information about timing of lek use by radiocollared fallow deer (**predictions 1.1, 1.2, and 2**, Table 1), including the number of individual visits (**predictions 1.3, 1.4**).

### Discontinuous and continuous radiotracking of deer

From April 1997 to December 2003, VHF-radiocollared males and females were monitored by discontinuous radiotracking (12–18 monthly fixes, homogenously distributed over day and night). We calculated locations by triangulation [34]. Seasons were defined as follows: winter (Dec.–Feb.), spring (Mar.–May), summer (Jun.–Aug.), and autumn (Sept.–Nov.). During autumn, i.e. the mating season, the monitoring effort significantly increased (1 fix every 12 h for both sexes from late August to November). To decrease the likelihood of a lek visit by a monitored deer being missed, special radiotracking teams used to patrol the Estate every hour at night and check the presence of deer with radiocollars in the two leks. From April 1997 to December 2003, 15,810 male fixes and 12,451 female fixes were collected using discontinuous radiotracking.

During the mating season from 1997 to 2003, females' daily movements at dawn and dusk (a.k.a. routes between night and day feeding areas) were also monitored using continuous radiotracking (1 fix every 15 minutes). When a female route ended inside the lek (namely female route to the lek), continuous radiotracking was maintained (6–36 hours) until the female left the lek and went back to her usual feeding areas (namely female route from the lek). Continuous sessions carried out at dawn and dusk were randomly distributed among all monitored females during the pre-rut, the rut, and the post rut periods. At the end of this research, 943 female routes were recorded.

### Male marking activities (visual references for dominance) outside the lek SG

In the period 1997–2003, from late August to late November, the same observer (SC) walked a 2,590 m transect around the lek SG every 3–4 days. Each visual/olfactory reference for dominance (i.e. trashing on the vegetation or scraping on the ground made by bucks [36]) observed along the transect was mapped onto a digitized 1∶2000 map of the lek area. This kind of data allowed us to gather further information about timing of male visits to the lek (**prediction 2**, Table 1)

### Data analyses

All data collections and analyses on lek activities and radiocollared deer refer to the south side of the San Rossore Estate, where the lek SG was located [32], [37], unless otherwise stated. Data collected in the northern side of the study area, where the lek FO was located, were specifically used to test those predictions that imply the occurrence of more than one lek within the same fallow deer population.

We combined observational data on the two leks with radiotracking data to determine lek use by radio-collared males and females (**predictions 1.1–1.4, and 2**, Table 1), *i.e.*, i) date of arrival to and departure from the lek, ii) number of individual lek visits by females, and iii) number of different leks visited by females.

We computed the size of 374 female seasonal home ranges (winter, spring, summer, and autumn) using the Ranges VI software [38]. Home ranges and home range centres were estimated using the 90% Kernel method [39], [40]. The linear distance between lek centre and home range centres was estimated using ArcGis 9.2. Using R 2.14 [41] (lme4 package, lmer function), we modelled the variation of log-transformed seasonal home range sizes using a linear mixed-effect model (LMEM [42]) with season included as fixed factor, and individual identity and year as random factors to avoid pseudoreplication of data [43]. We adopted the same LMEM approach to model seasonal variation of the distance between female home range centres and lek centres. These analyses were aimed to test **prediction 4** (Table 1) related to the size of home ranges and their position with respect to the lek.

To test our **prediction 5.1** (Table 1), maximum overlap areas of female home ranges recorded during the autumnal mating season (namely hotspots) were computed using ArcGis 9.2. Hotspots were defined as the overlap area between home ranges of at least 3 females belonging to different social units. The social unit was defined as the group of adult females and fawns moving together along daily routes at dawn and dusk; females fitted with radiocollars not belonging to the same social unit were never relocated together during continuous radiotracking sessions. We estimated the distance from and the overlap with the lek area for each hotspot (**prediction 5.1**). Female hotspots were computed on a yearly basis. We also estimated the number of leks included within female home ranges (**prediction 3.1**), and we calculated the average diameter of a female home range during the autumnal mating season (**prediction 3.2**).

Female routes collected at dawn and dusk using continuous radiotracking were analysed using the patch Animal Movements SA v 2.04 [44]. We calculated the following variables for each route: (i) linear distance between the lek centre and the nearest fix of the route (in meters), (ii) total distance covered during the route (in meters), and (iii) average speed meters/min. Variables ii) and iii) were also computed for female routes to and from the lek.

To test our **predictions 5.2** (Table 1), we modelled the variation of linear distance between lek centre and the nearest fix of female routes by fitting a LME model with sub-period (pre-rut, rut, post-rut) and period of the day (dawn, dusk) included as fixed factors, and deer identity and year as random factors.

To compare usual female daily routes with those to and from the lek (**prediction 6**), we fitted two LME models with total distance covered (log-transformed) and speed of routes as dependent variables, respectively, movement type (usual routes at dawn and dusk, or routes to and from the lek) as fixed factor, and deer identity and year as random factors.

### Position of lek SG with respect to female travel costs and predation risk

The southern sector of the study site (*i.e.*, where the lek SG is located) was subdivided into 300×300 grid squares equivalent in size to the area of lek SG using ArcGis 9.2. All grid squares were assumed to be a location of a hypothetical lek.

First, we calculated the linear distance (*i.e.*, the travel cost) required by a female to go from the centre of a female hotspot to each hypothetical lek within 300×300 grid squares. Hypothetical leks were ranked based on travel costs required by females to reach them.

Second, we estimated the linear distance between the eastern disturbed sector (*i.e.*, the sector with the highest predation risk [35]) and each hypothetical lek. Hypothetical leks were ranked based on the degree of predation risk, based on the assumption that the higher the distance from the disturbed sector, the lower the predation risk perceived by deer [33], [34].

Third, we combined travel costs with the degree of perceived predation risk for each hypothetical lek, we identified areas with the best balance between the two factors, and we verified where the actual lek SG is located within a GIS framework.

## Results

### Lek use by females and males (occurrence and timing)

Lek use by monitored females and males is reported in Table 2. Almost all females and males used the lek during the rut. Females commonly visited the lek only once during the same rut, rarely twice (only 6 out 81 cases from 1997 to 2003, Table 2) and never three times. Mean time interval between consecutive lek visits for individual females who visited the lek more than once was 5 days (range 1–10 days). No collared females visited more than one lek during the same rut (Table 2).

**Table 2:** Lek use in male and female fallow deer.

	Overall study period (1997–2003)	Rut 1997	Rut 1998	Rut 1999	Rut 2000	Rut 2001	Rut 2002	Rut 2003
**1.1 -** Percentage of females that visited the lek during the rut	**89.0% (female cases over total 81/91)**	70% (7/10)	90% (9/10)	100% (8/8)	93% (14/15)	93% (14/15)	87% (13/15)	89% (16/18)
**1.2 -** Percentage of males that visited the lek during the rut	**95.6% (male cases over total 87/91)**	100% (9/9)	100% (12/12)	87.5% (21/24)	100% (16/16)	100% (15/15)	90% (9/10)	100% (5/5)
**1.3 -** Number of visits to the lek per female^1^ during the rut	**1.1±0.3 visits (mean ± SD; female cases n = 81)**	1.1±0.4 (n = 7)	1.3±0.5 (n = 9)	1.1±0.4 (n = 8)	1.2±0.4 (n = 14)	1.0±0 (n = 14)	1.0±0 (n = 13)	1.0±0 (n = 16)
**1.4 -** Number of leks visited by each female during the rut	**1.0±0 lek visited (mean ± SD; female cases n = 81)**	1.0±0 (n = 7)	1.0±0 (n = 9)	1.0±0 (n = 8)	1.0±0 (n = 14)	1.0±0 (n = 14)	1.0±0 (n = 13)	1.0±0 (n = 16)

1 Females that did not visit a lek were excluded.

Lek use by radiocollared male and female fallow deer in the San Rossore Estate during 7 consecutive rutting periods. Lek use was computed combining direct lek observations with spatial data gathered by continuous and discontinuous radiotracking.

Males of this fallow deer population began to use the lek in early September, as shown by the occurrence of marking activities recorded around the lek (Fig. 1, upper panel), *i.e.*, about one month before the first copulation observed there. This was confirmed by radiotracking data, with radiocollared bucks arriving at the lek from late August to mid-September (Fig. 1, lower panel). Radiocollared females began to visit the lek in October, right after the observation of the first mating (Fig. 1, lower panel). Females showed up in the lek about one month later than bucks (Fig. 1, lower panel). Females stopped visiting the lek in the second half of October, when males also began to gradually leave the mating area (Fig. 1, lower panel).

**Figure 2:**
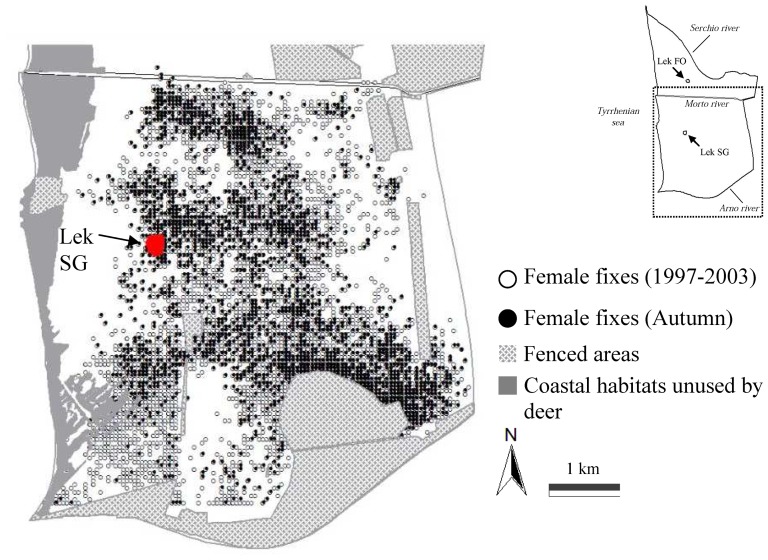
Spatial relocations of male and female fallow deer. Spatial distribution of female fallow deer fixes (white dots) recorded during 7 consecutive years in San Rossore. Relocations collected during the autumn were those represented by black dots. The lek area, fenced areas (not available to deer), and coastal habitats unused by deer (*i.e.* maritime pine woods, degraded coastal zone, dune vegetation) were reported in the map.

Although we made direct observations and monitored the presence of radiocollared deer in both leks, we focused our 7-year radiotracking program in the southern sector of the San Rossore Estate, where lek SG was located (Fig. 2). The two fallow deer sub-populations (northern and southern side of Morto River, Fig. 2) had both 1 lek site. In regard to lek use, from 1997 to 2003 no monitored deer of either sex visited 2 leks during the same year. In regard to deer movements across the two sub-population, two females and one male monitored in the southern side of the study area used the lek FO in the northern side.

### Relationships among lek position and female home ranges and movements

Female home ranges in autumn never included 2 leks (0 out 91 cases). Only 20 female autumn home ranges (21%) included 1 lek from 1997 to 2003 (in 1997: 0/10 females; in 1998: 0/10; in 1999: 0/8; in 2000: 4/15; in 2001: 8/15; in 2002: 5/15; in 2003: 3/18). Mean size of female autumn home ranges was 319.48 ha (SE = ±249.47 ha). Assuming a circular shape of a female home range (*sensu* [23]), the average home range diameter in autumn was 2017 m, more than 1 km shorter than the distance between the two leks of the San Rossore Estate, *i.e.*, 3200 m.

We recorded a significant variation of female home range sizes between seasons (LME model, Table S1). Female home ranges did not differ in size (*i.e.*, overlapping 95% CIs, see Table S1) during winter (147.37±12.32 ha), spring (191.25±13.67 ha), and summer (138.50±10.99 ha), while home ranges significantly increased in size during the autumn mating season (319.48±26.30 ha; p_LRT_<0.001 in all cases, Table S1). We also found a significant seasonal variation of the linear distance between home range centres and lek centres (Table S2). The shortest distance was recorded in summer (1845.65±71.17 m; Table S2) but not in the autumnal mating season (2254.43±108.05 m), which did not differ to those recorded in winter (2387.13±104.19 m) or spring (2187.51±81.67 m) (p_LRT_ = 0.121 and p_LRT_ = 0.212, respectively; Table S2).

**Figure 3:**
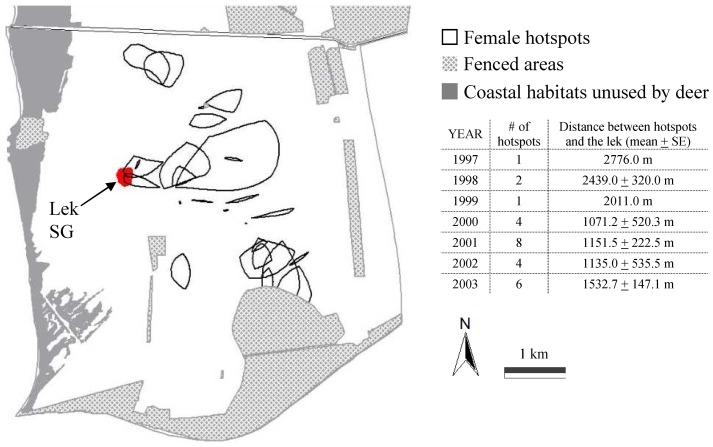
Location of female hotspots with respect to lek position. Spatial location of high female fallow deer traffic areas (hotspots) recorded in autumn from 1997 to 2003 in San Rossore. Hotspots were defined as the maximum overlap areas occurring among at least 3 home ranges (Kernel 90% isopleths) of females belonging to different social units. The lek area, fenced areas (not available to deer), and coastal habitats unused by deer (*i.e.* maritime pine woods, degraded coastal zone, dune vegetation), and the distance between the lek and hotspots were reported in the map.

Female locations collected from 1997 to 2003 were shown in Fig. 2. Female hotspots were reported in Fig. 3. Hotspots commonly were more than 1 km distant (mean ± SE: 1537.0±146.41 m) from the lek centre (Fig. 3), with the exception of two hotspots (total hotspots n = 26) that overlapped the lek area (Fig. 3). This was the case in 2001, with a hotspot overlapping the home ranges of 7 females, and in 2002 (5 females) (Fig. 3).

In regard to the linear distance between the closest fix to the lek of female daily routes in autumn, no difference was found between routes recorded at dawn or dusk (LME model p_LRT_ = 0.683, Table S3), whereas a significant variation of such a distance was found among pre-rut, rut and post-rut (Table S3). Indeed, lower distances between the lek centre and the nearest fix of female routes were recorded during the rut (mean ± SE 1510.66±55.75 m), if compared to the pre-rut (1675.03±44.21 m) and post-rut (1908.56±56.60 m) (p_LRT_ = 0.001 and p_LRT_<0.001, respectively; Table S3). Therefore, even if routes recorded during the rut were the closest to the lek, they were still more than 1.5 km distant from it. No female routes crossed the lek during pre-rut, rut, and post-rut, with the only exception of those specific routes walked to go to the lek, *i.e.*, with final location of the route located in the lek area.

We compared usual daily routes walked by females at dawn or dusk with those directed to or starting from the lek (LME model, Table S4). The total distance covered at dawn or dusk by females during their daily usual routes (1403.78±31.29 m) was significantly lower than that covered either to visit (2783.63±241.22 m) or to leave the lek (2748.23±181.41 m) (p_LRT_<0.001 in both cases; Table S4). Female routes to and from the lek were walked more quickly than usual daily routes (LME model p_LRT_ = 0.012 and p_LRT_<0.001, respectively; Table S5). Females walked to and from the lek at an average speed of 12.0±1.04 m/min, whereas daily routes were walked at lower speed, both at dawn (9.41±0.26 m/min) and dusk (9.20±0.31 m/min) (Table S5).

### Position of lek SG with respect to female travel costs and predation risk

**Figure 4:**
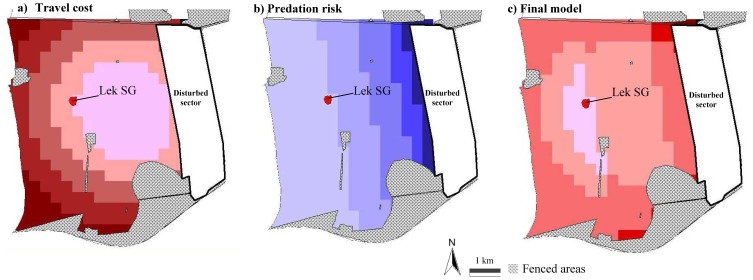
The handy location of lek SG: balancing the cost of mate assessment against predation risk. Model of the southern side of the study area showing how it was subdivided by 300×300 m grid squares, each of them representing a hypothetical lek. The travel cost (linear distance) required by females to move from hotspot centres to each hypothetical lek was reported in the left map (**a**), where darker colours indicate hypothetical leks that require higher travel costs to be reached by females. The predation risk of each hypothetical lek was computed as the linear distance from the disturbed sector and was shown in the central map (**b**), where lighter colours indicate hypothetical leks with lower predation risk by humans. The right map (**c**) represents the final spatial model which combines travel cost and predation risk values. Lighter areas represent those hypothetical leks with the best combination of low travel cost and low predation risk. Lek SG is located right in this area of the study site.

The travel cost (linear distance) required by females to move from hotspot centres to each hypothetical lek was reported in Fig. 4a; darker colours indicate hypothetical leks that require higher travel costs to be reached by females. The predation risk of each hypothetical lek was computed as the linear distance from the disturbed sector and was shown in Fig. 4b; lighter colours indicate hypothetical leks with lower predation risk by humans. Figure 4c represents the final spatial model that combines travel costs with predation risk; lighter areas represent those hypothetical leks with the best combination of low travel cost and low predation risk. Lek SG is located right in this area of the study site.

## Discussion

The majority of females visited the lek during the rut including those without a lek within their home range (**prediction 1.1**) confirming that females strongly prefer large clumps of males that provide high mate choice opportunities [22]. This was in agreement with the female preference model [1], [13], [22]. The majority of males used the lek (**prediction 1.2**), *i.e.*, the place that can guarantee the highest mating success, at least for those males that obtain a dominance status [8]. Again, in accordance with the female preference model [1], [13], [22], females visited only one lek (**prediction 1.4**), and generally visited it just once (**prediction 1.3**). The second visit of females to the same lek that was recorded in few cases occurred on average 5 days after the first visit. It is unlikely that the second visit represented the female attempt to mate again if fecundation had not occurred during the previous visit, because there was not enough time for the animal to return to oestrus and for ovulation to occur again. In fact, the length of the oestrus cycle in fallow deer is about 24–28 days [36]. Multiple visits could favour mate quality assessment, and further research is needed to address this topic.

We showed that males arrived at the lek well before the arrival of females (**prediction 2**), in contrast to what predicted by the hotspot model [1]. During the month spent in the lek area before the arrival of females, males establish and mark territories within the lek [36], and create a number of scent marking stations around the lek that are individual references for dominance [36], [45]. Dominance hierarchy among fallow bucks is mainly established through non-contact interactions that occur during the pre-rut period [24], [46]. Visual and/or olfactory marking activities are supposed to be important for male status signalling in male-male interaction, as well as for mate choice by females [45].

The lek SG was not located in an area typically used by females (**prediction 5.1**) and was spaced more than an average female home range diameter apart from the other lek (**prediction 3.2**). This result again is not consistent with the predictions of the hotspot model, which predicts more than one lek per female home range [1], whereas it was only one in our study case (**prediction 3.1**). Indeed, we showed that females increased home range sizes to reach the lek (**prediction 4**). Our results suggest that females leave their usual foraging areas to reach the lek with faster, more direct, and longer routes if compared to usual daily movements recorded at dawn and dusk (**prediction 5.2, prediction 6**). Together, these results do not support the prediction of the hotspot model [13], [23] that routes leading to the lek should be even shorter than usual daily routes. Leks seem to be more commonly overlapping with female hotspots in birds rather than in ungulates [1], [5], [12], [47], [48].

Both leks were located far from the disturbed sector with the highest predation risk for deer [32], [35], supporting the predictions of the predator avoidance model (**prediction 5.1, prediction 6**). Leks were used by the majority of animals (**predictions 1.1, 1.2**), thus increasing the likelihood of reduced predation risk due to dilution effect [1], [11].

Our results, therefore, supported all the predictions of the female preference model and of the predator avoidance model as the most likely candidates able to explain the persistence of the lek SG in that specific location (Table 1, 2, 3). Predictions of the hotspot model, instead, were not supported (Table 2–3).

**Table 3:** Supported predictions of models on lek formation.

		Models' predictions		
	Field data (1997–2003)	Female preference model	Hotspot model	Predator avoidance model
**LEK VISITS (OCCURRENCE AND TIMING)**	**1.1**	SUPPORTED	NOT SUPPORTED	SUPPORTED
	**1.2**	SUPPORTED	-	SUPPORTED
	**1.3**	SUPPORTED	NOT SUPPORTED	*-*
	**1.4**	SUPPORTED	NOT SUPPORTED	*-*
	**2**	SUPPORTED	NOT SUPPORTED	*-*
**RELATIONSHIP BETWEEN POSITION OF LEKS AND FEMALE HOME RANGES AND MOVEMENTS**	**3.1**	SUPPORTED	NOT SUPPORTED	*-*
	**3.2**	SUPPORTED	NOT SUPPORTED	*-*
	**4**	SUPPORTED	NOT SUPPORTED	*-*
	**5.1**	SUPPORTED	NOT SUPPORTED	SUPPORTED
	**5.2**	SUPPORTED	NOT SUPPORTED	-
	**6**	SUPPORTED	NOT SUPPORTED	SUPPORTED

Predictions of models on lek formation that were supported by field data in the lekking fallow deer population of San Rossore (see Table 1 for details on field data and models' predictions).

Different models of lek formation and persistence could be valid in different phases of the temporal evolution of mating arenas. Bradbury and Gibson [13] claimed that leks may be initially spaced according to the hotspot model, but female preference for certain leks could have contributed to the disappearance of interstitial leks. On support of this hypothesis, we remind that a further lek (lek Macchia Capraia MC) was present until late 1980s in San Rossore [7], when lek FO and lek SG were already known lekking sites in this study area. The lek MC was indeed about 1 km east from lek SG [7], a certainly lower distance than that recorded in our study site for female home range diameters. Lek MC was also overlapping a female traffic hotspot during the rut [7]. The hotspot model was proposed as a likely explanation for the persistence of lek MC [7], even though we must acknowledge that such conclusion was based on direct observations of females crossing the lek at twilight, with no support from radiotelemetry data. In 1987, habitat modification (mainly logging) presumably affected areas near the lek MC that were used by females as travel routes, and, consequently, this lek disappeared within the 3 following years [7]. Meanwhile, human disturbance and human predation pressure have increased in the eastern sector of the study site since early 90s [33], [35], creating the new ecological conditions showed in our research. The current position of lek SG strongly supports the female preference model and the predator avoidance model, but the existence in the past of the lek MC partly confirms the vision of Bradbury and Gibson [13] on the different phases of the temporal evolution of mating arenas.

The main drawback of our research is that we cannot shed light on the factors that may have been responsible for the formation of a lek. Lek (MC) was present in the 1980s [7], and the presence of other lekking sites prior to the beginning of this long-term research program has been reported by local park wardens. Multiple factors might have concurred to lek formation, also depending on different ecological conditions of the past, *e.g.*, when different deer spatial behaviour could be expected due to the absence of human disturbance before the 1980s [33].

The key point of this research, instead, is a clear evidence on which selective pressures guarantee the persistence of the lek SG in that specific position over the 7 years of this study, *i.e.*, female preference and predator avoidance. This is the first time that this evidence has been shown for ungulates with empirical observation and radiotracking field data. Our results confirm the vision of Bradbury [26] and Bradbury & Gibson [13] that suggested i) that female preference for larger leks would cause males to cluster until there is a single lek per population or per female home range, and ii) leks should thus be spaced an average female home range diameter apart (e.g., only one lek within a female home range), and each female should visit only one lek. Starting from 3 lekking sites in the 1980s, Bradbury and Gibson's vision could explain the persistence of 1 lek site per deer sub-population in our study site (*i.e.*, lek SG and lek FO for the southern and northern deer sub-populations, respectively).

Multiple factors are thought to be responsible for the formation and the persistence of a lek site, the combination of which depends on local ecological conditions. In our study, lek SG clearly is maintained by female preference and predator avoidance. However, other leks could form on account of different selective pressures, as confirmed by contrasting studies available in literature. Indeed, the evolution and maintenance of lek-breeding behaviour remain unclear and the subject of remarkable controversy [49], [50]. According to Höglund and Alatalo [1], it is not possible to find a unique explanation for lek evolution. Lekking is likely the most difficult mating strategy to be explained within an evolutionary framework [1], [11]. Given the differences in the ecology and life histories of lekking species, multiple explanations of lek formation are reasonably coexisting across different taxa. For instance, the hotshot model is the best explanation for lek formation in marine iguanas (*Amblyrhynchus cristatus*) [51]. Westcott [52], in a study on ochre-bellied flycatcher (*Mionectes oleaginous*), and Jones & Quinnell [53], in sandfly (*Lutzomyia longipalpis*), supported the hotspot model as a possible mechanism promoting lek formation. In a more recent paper, Young et al. [16] strongly supported the female preference model in bower-building cichlid fish (*Nyassachromis microcephalus*). In a study dealing with three ungulate species, Balmford et al. [12] suggested that, while the hotspot model may explain broad patterns of male dispersion, further mechanisms are needed to generate the extent of territory clustering seen at leks. Clutton-Brock et al. [21] clearly showed the importance of harassment in favouring lekking in fallow deer, at least when high population densities occur, although the avoidance of harassment by females was considered unlikely to explain lek evolution in topi [54], [55] and Kafue lechwe antelopes *Kobus leche kafuensis* [56].

Multiple factors could be responsible for the persistence of leks in ungulates, even within the same population. We believe that a single general explanation of lek formation and persistence could hardly exist, because it might depend on a combination of selective pressures that is based on local ecological conditions and favours lek persistence. Lek SG current ‘handy’ position minimizes travel costs and maximizes predator avoidance at the sub-population level. That a lek should be ‘handy’ was already suggested by Oring [57] many years ago: ‘*males ought to display at the site having the lowest cumulative distance from the activity centres of all females of the population*’. The female preference model explains why there is only one big lek in the southern subpopulation of San Rossore, while the ‘handy’ idea explains why it is located in that position. Lek FO could be handy for the deer sub-population using the northern side of the Estate, by virtue of its central location and wide distance from the high predation risk area (Fig. 2), such as recorded for lek SG. Although this hypothesis would need another intensive radio-tracking program in the northern side of the Estate to be supported.

## Supporting Information

Table S1 Parameters estimated by the linear mixed model predicting the variation of seasonal home range sizes in female fallow deer.
(DOCX)

Table S2 Parameters estimated by the linear mixed model predicting the seasonal variation of the distance between female home range centres and lek centres in fallow deer.
(DOCX)

Table S3 Parameters estimated by the linear mixed model predicting the variation of the linear distance between lek centre and the nearest fix of female fallow deer routes during sub-periods (pre-rut, rut, post-rut) and period of the day (dawn, dusk).
(DOCX)

Table S4 Parameters estimated by the linear mixed model predicting the variation of the distance (ln-transformed) walked by female fallow deer during usual routes (recorded at dawn and dusk) and during the routes to and from the lek.
(DOCX)

Table S5 Parameters estimated by the linear mixed model predicting the variation of speed in female fallow deer while walking usual routes (at dawn and dusk) and those to and from the lek.
(DOCX)
