# Supplementary material for: Female Preference and Predation Risk Models Can Explain the Maintenance of a Fallow Deer (Dama dama) Lek and Its ‘Handy’ Location
Source: PLoS One. 2014 Mar 5;9(3):e89852. doi: 10.1371/journal.pone.0089852 (PMC3943860; doi:10.1371/journal.pone.0089852)
Supplement: Table S1 — Parameters estimated by the linear mixed model predicting the variation of seasonal home range sizes in female fallow deer. (DOCX) [file pone.0089852.s001.docx]

**Table S1. Parameters estimated by the linear mixed model predicting the variation of seasonal home range sizes in female fallow deer.**

| Fixed effects | ***β*** | ***SE*** | **lower 95% *CI*** | **upper 95% *CI*** | ***t*** | ***p_LRT_*** |
| --- | --- | --- | --- | --- | --- | --- |
| *intercept* | 5.44 | 0.13 | 5.19 | 5.68 | 43.0 | <0.001 |
|  |  |  |  |  |  |  |
| spring | -0.46 | 0.10 | -0.66 | -0.26 | -4.6 | <0.001 |
| summer | -0.78 | 0.10 | -0.98 | -0.58 | -7.7 | <0.001 |
| winter | -0.77 | 0.10 | -0.97 | -0.56 | -7.3 | <0.001 |
| autumn | 0^a^ | - | - | - | - | - |

|  |
| --- |

^a^this parameter is set to zero because it is redundant

_pLRT_: p value based on likelihood ratio test for fixed-effects terms. Autumn is the reference category.

Dependent variable: seasonal home range sizes (ln-transformed)

Random effects: deer identity and year

Number of observations: 374

Number of females: 30

Number of years: 7
