# Supplementary material for: Female Preference and Predation Risk Models Can Explain the Maintenance of a Fallow Deer (Dama dama) Lek and Its ‘Handy’ Location
Source: PLoS One. 2014 Mar 5;9(3):e89852. doi: 10.1371/journal.pone.0089852 (PMC3943860; doi:10.1371/journal.pone.0089852)
Supplement: Table S2 — Parameters estimated by the linear mixed model predicting the seasonal variation of the distance between female home range centres and lek centres in fallow deer. (DOCX) [file pone.0089852.s002.docx]

**Table S2. Parameters estimated by the linear mixed model predicting the seasonal variation of the distance between female home range centres and lek centres in fallow deer.**

| Fixed effects | ***β*** | ***SE*** | **lower 95% *CI*** | **upper 95% *CI*** | ***t*** | ***p_LRT_*** |
| --- | --- | --- | --- | --- | --- | --- |
| *intercept* | 2287.1 | 146.0 | 2000.9 | 2573.3 | 15.6 | <0.001 |
|  |  |  |  |  |  |  |
| spring | -92.8 | 74.5 | -238.9 | 53.2 | -1.2 | 0.212 |
| summer | -423.9 | 74.8 | -570.5 | -277.3 | -5.6 | <0.001 |
| winter | 119.3 | 76.9 | -31.5 | 270.1 | 1.5 | 0.121 |
| autumn | 0^a^ | - | - | - | - | - |

|  |
| --- |

^a^this parameter is set to zero because it is redundant

_pLRT_: p value based on likelihood ratio test for fixed-effects terms. Autumn is the reference category.

Dependent variable: distance between female home range centres and lek centres

Random effects: deer identity and year

Number of observations: 374

Number of females: 30

Number of years: 7
