# Supplementary material for: Female Preference and Predation Risk Models Can Explain the Maintenance of a Fallow Deer (Dama dama) Lek and Its ‘Handy’ Location
Source: PLoS One. 2014 Mar 5;9(3):e89852. doi: 10.1371/journal.pone.0089852 (PMC3943860; doi:10.1371/journal.pone.0089852)
Supplement: Table S3 — Parameters estimated by the linear mixed model predicting the variation of the linear distance between lek centre and the nearest fix of female fallow deer routes during sub-periods (pre-rut, rut, post-rut) and period of the day (dawn, dusk). (DOCX) [file pone.0089852.s003.docx]

**Table S3. Parameters estimated by the linear mixed model predicting the variation of the linear distance between lek centre and the nearest fix of female fallow deer routes during sub-periods (pre-rut, rut, post-rut) and period of the day (dawn, dusk).**

| Fixed effects | ***β*** | ***SE*** | **lower 95% *CI*** | **upper 95% *CI*** | ***t*** | ***p_LRT_*** |
| --- | --- | --- | --- | --- | --- | --- |
| *intercept* | 1562.23 | 147.70 | 1272.75 | 1851.71 | 10.58 | <0.001 |
|  |  |  |  |  |  |  |
| pre-rut | 179.13 | 51.85 | 77.50 | 280.76 | 3.46 | 0.001 |
| post-rut | 410.32 | 56.64 | 299.31 | 521.33 | 7.25 | <0.001 |
| rut | 0^a^ | - | - | - | - | - |
|  |  |  |  |  |  |  |
| dusk | -17.00 | 41.78 | -98.89 | 64.89 | -0.41 | 0.683 |
| dawn | 0^a^ | - | - | - | - | - |

|  |
| --- |

^a^this parameter is set to zero because it is redundant

_pLRT_: p value based on likelihood ratio test for fixed-effects terms. Rut and dawn are the reference categories.

Dependent variable: linear distance between lek centre and the nearest fix of female routes

Random effects: deer identity and year

Number of observations: 828

Number of females: 27

Number of years: 7
