# Supplementary material for: Female Preference and Predation Risk Models Can Explain the Maintenance of a Fallow Deer (Dama dama) Lek and Its ‘Handy’ Location
Source: PLoS One. 2014 Mar 5;9(3):e89852. doi: 10.1371/journal.pone.0089852 (PMC3943860; doi:10.1371/journal.pone.0089852)
Supplement: Table S4 — Parameters estimated by the linear mixed model predicting the variation of the distance (ln-transformed) walked by female fallow deer during usual routes (recorded at dawn and dusk) and during the routes to and from the lek. (DOCX) [file pone.0089852.s004.docx]

**Table S4. Parameters estimated by the linear mixed model predicting the variation of the distance (ln-transformed) walked by female fallow deer during usual routes (recorded at dawn and dusk) and during the routes to and from the lek.**

| Fixed effects | ***β*** | ***SE*** | **lower 95% *CI*** | **upper 95% *CI*** | ***t*** | ***p_LRT_*** |
| --- | --- | --- | --- | --- | --- | --- |
| *intercept* | 7.03 | 0.12 | 6.81 | 7.26 | 60.88 | <0.001 |
|  |  |  |  |  |  |  |
| route from the lek | 0.84 | 0.08 | 0.68 | 0.99 | 10.80 | <0.001 |
| route to the lek | 0.83 | 0.13 | 0.57 | 1.08 | 6.31 | <0.001 |
| usual route | 0^a^ | - | - | - | - | - |

|  |
| --- |

^a^this parameter is set to zero because it is redundant

_pLRT_: p value based on likelihood ratio test for fixed-effects terms. Usual route is the reference category.

Dependent variable: total distance (ln-transformed) walked by females during routes

Random effects: deer identity and year

Number of observations: 913

Number of females: 27

Number of years: 7
