# Supplementary material for: Female Preference and Predation Risk Models Can Explain the Maintenance of a Fallow Deer (Dama dama) Lek and Its ‘Handy’ Location
Source: PLoS One. 2014 Mar 5;9(3):e89852. doi: 10.1371/journal.pone.0089852 (PMC3943860; doi:10.1371/journal.pone.0089852)
Supplement: Table S5 — Parameters estimated by the linear mixed model predicting the variation of speed in female fallow deer while walking usual routes (at dawn and dusk) and those to and from the lek. (DOCX) [file pone.0089852.s005.docx]

**Table S5. Parameters estimated by the linear mixed model predicting the variation of speed in female fallow deer while walking usual routes (at dawn and dusk) and those to and from the lek.**

| Fixed effects | ***β*** | ***SE*** | **lower 95% *CI*** | **upper 95% *CI*** | ***t*** | ***p_LRT_*** |
| --- | --- | --- | --- | --- | --- | --- |
| *intercept* | 9.18 | 0.98 | 7.26 | 11.10 | 9.37 | <0.001 |
|  |  |  |  |  |  |  |
| route from the lek | 3.03 | 0.74 | 1.58 | 4.49 | 4.08 | <0.001 |
| route to the lek | 3.17 | 1.26 | 0.70 | 5.63 | 2.51 | 0.012 |
| usual route | 0^a^ | - | - | - | - | - |

|  |
| --- |

^a^this parameter is set to zero because it is redundant

_pLRT_: p value based on likelihood ratio test for fixed-effects terms. Usual route is the reference category.

Dependent variable: speed of females to walk routes

Random effects: deer identity and year

Number of observations: 913

Number of females: 27

Number of years: 7
